# Size and Shape of Associations of OGTT as Well as Mediating Effects on Adverse Pregnancy Outcomes Among Women With Gestational Diabetes Mellitus: Population-Based Study From Southern Han Chinese

**DOI:** 10.3389/fendo.2020.00135

**Published:** 2020-03-17

**Authors:** Zixing Zhou, Gengdong Chen, Dazhi Fan, Jiaming Rao, Pengsheng Li, Shuzhen Wu, Dongxin Lin, Huiting Ma, Shaoxin Ye, Huishan Zhang, Xiuyin Shen, Yingchun Wan, Xin Luo, Dongmei Suo, Xiaoling Guo, Zhengping Liu

**Affiliations:** ^1^Foshan Fetal Medicine Research Institute, Foshan Women and Children Hospital Affiliated to Southern Medical University, Foshan, China; ^2^Department of Obstetrics, Foshan Women and Children Hospital Affiliated to Southern Medical University, Foshan, China

**Keywords:** gestational diabetes mellitus, OGTT, adverse pregnancy outcomes, restricted cubic spline, mediating effect

## Abstract

**Objective:** To explore the size and shape association of OGTT values with adverse pregnancy complications among women with gestational diabetes mellitus (GDM) in Southern Han Chinese population and further analyze their mediating effects with maternal age in outcomes.

**Methods:** 6,861 women with GDM were included in the study. Logistic regression was used to identify the correlations between OGTT values and adverse pregnancy outcomes of GDM. Restricted cubic spline nested logistic regression was conducted to investigate potential non-linear and linear associations. Mediating effect among maternal age, OGTT and adverse outcomes were explored.

**Results:** Women with GDM had a mean age of 31.83, and 24.49% had advanced maternal age (≥35 years). In logistic regression with adjustment, compared with lower OGTT0 (<5.1 mmol/L), GDM patients with higher OGTT0 (≥5.1 mmol/L) exhibited 1.891 (95% CI: 1.441–2.298, *P* < 0.001), 1.284 (1.078–1.529, *P* = 0.005), 1.285 (1.065–1.550, *P* = 0.009), and 1.302 (1.067–1.590, *P* = 0.010) times increased risk of hypertensive disorders of pregnancy (HDP), preterm, neonatal hyperbilirubinemia, and macrosomia, respectively. GDM patients with higher OGTT1 (≥10 mmol/L) had only found to exhibited 1.473-fold (1.162–1.867, *P* = 0.001) increasing risk of HDP than those with lower OGTT1 (<10 mmol/L). No adverse outcome was identified to associate with higher OGTT2 (≥8.5 mmol/L). Linear relationships (non-linear *P* > 0.05) were observed between OGTT0 and HDP, preterm, neonatal hyperbilirubinemia, and macrosomia in both maternal age groups (<35 and ≥35 years). Non-linear associations of OGTT1 with incidence of HDP, preterm, and neonatal hyperbilirubinemia were detected in GDM patients younger than 35 years (non-linear *P* = 0.037, *P* = 0.049, *P* = 0.039, respectively), rising more steeply at higher values. Similar non-linearity was noted for OGTT2 with HDP in older patients. All OGTT values had significant mediating effects on some special complications caused by higher age.

**Conclusion:** Higher fasting plasma glucose was more strongly linked to adverse pregnancy outcomes among GDM patients. Both linearity and Non-linearity of associations between glucose and complications should be taken into account. A careful reconsideration of GDM with hierarchical and individualized management according to OGTT is needed.

## Introduction

The prevalence of gestational diabetes mellitus (GDM) has been increasing globally with increasing BMI and reproductive age in women (1, 2). The incidence of GDM in China was approximately 17.5% (3). GDM arises because of insulin resistance or diminish action of insulin due to hormone production by the placenta. Other risk elements for GDM include older age, obesity, and so on (1). GDM is associated with adverse maternal, fetal, and neonatal outcomes in the short term and adverse health impacts on women themselves and offspring in the long term. Women with GDM are at greater risk of high blood pressure, infection, and macrosomia which might result in difficult and risky delivery, while fetal or neonatal complications are more likely to include hypoglycemia, hypocalcaemia, and respiratory distress (4).

Seventy-five gram OGTT test performed between 24 and 28 weeks of gestation is the diagnosis standard of GDM. Treatment with oral agent or insulin and interventions of lifestyle and diet are often performed on GDM patients according to the test result, and the knowledge of these may estimate and further improve the effect of the interventions. The Hyperglycaemia and Adverse Pregnancy Outcomes (HAPO) study demonstrated continuously increased relationship between adverse pregnancy outcomes and maternal glucose without specific threshold (5). Other studies reported linear continuous association of glycemic markers with diabetic risk (6, 7). Only a limited number of articles showed a non-linear relation between blood glucose and diabetes (8, 9). However, the precise correlations between three values' characteristics of OGTT at different points of time and adverse outcomes among women with GDM still remain ambiguous. As glucose value of OGTT is a continuous variable, there might be non-linear or curvilinear relationship between OGTT and adverse outcomes. It is unclear whether different size and shape of the curve of continuous values of fasting plasma glucose, 1-h plasma glucose or 2-h on OGTT represent a different profile of risk for adverse outcomes and whether they represent different or specific risks in GDM, especially in Chinese GDM patients.

As China has abolished its one-child policy and implemented the two-child policy, the proportion of an advanced maternal age continues to increase. Advance maternal age was identified as a risk factor for GDM (3, 10, 11). A Dose-response analysis reported a positive linear relationship between GDM risk and maternal age, and GDM risk of the Asian increased by 12.74% for each 1-year increase from 18 years (12). Women with age ≥35 years are commonly related to adverse pregnancy outcomes, and those aged 45 years old and older had the highest risk (13). The potential relationship among maternal age, glucose, and adverse outcomes is unclear and need further exploration.

A more particular knowledge of these relations can improve the development of diabetic individuation management strategies for GDM patients according to individual OGTT characteristics. Therefore, the aim of our study was to estimate the magnitude and shape of the association of OGTT results and the risk of adverse outcomes of among Southern Han Chinese GDM patients. Further, we sought to assess which OGTT parameter was best linked to specific adverse pregnancy complications, and to assess these associations according to age of GDM patients.

## Methods

### Study Population

In the study, all of participants were collected from Southern Medical University Affiliated Maternal & Child Health Hospital of Foshan city, Guangdong province, China from January 2015 to August 2018. According to the eligibility and exclusion criteria, 6,861 women with GDM were included in the study who were discharged after finishing the delivery hospitalizations (Figure S1). This hospital was a tertiary university-affiliated medical center. All participants were Chinese Han residents. The participants gave informed consent when they were about to leave the hospital after finishing the delivery hospitalizations. The Human Subjects Committee of the Southern Medical University Affiliated Maternal & Child Health Hospital of Foshan approved the study protocol. The eligibility criteria include women with GDM who had singleton pregnancy and had performed a 75 g OGTT test between 24 and 28 weeks of gestation. The exclusion criteria were those women who had pregestational diabetes mellitus (PGDM), malignant diseases or missing OGTT data. Once a pregnancy woman was diagnosed with GDM, obstetricians and nutritionists in our hospital can help her control her blood glucose levels through a healthy diet, gentle exercise, and blood glucose monitoring. In some cases, insulin may also be prescribed. All women with GDM didn't treated with oral hypoglycemic drugs in pregnancy.

### Data Collection and Definition

We collected the data retrospectively. When all participants finished their delivery hospitalizations, we submitted the information request form to the hospital and then searched the information with permission. In order to ensure participation's privacy, the sensitive information such as name, phone number, home address were excluded.

GDM is diagnosed by the International Association for Diabetes in Pregnancy Study Group's (IADPSG) criteria, i.e., having met any one of the cutoff points: fasting plasma glucose (FPG, OGTT0) ≥ 5.1 mmol/L (92 mg/dL) or 1-h PG (OGTT1) ≥10.0 mmol/L (180 mg/dL) or 2-h PG (OGTT2) ≥8.5 mmol/L (153 mg/dL) (14). Hypertensive disorders of pregnancy (HDP), includes gestational hypertension, preeclampsia, eclampsia, superimposed preeclampsia on chronic hypertension and chronic hypertension in pregnancy (15). Intrahepatic cholestasis of pregnancy (ICP) is diagnosed in women presenting with classical pruritus associated with liver dysfunction and raised total serum bile acids (TBA ≥ 10 μmol/L), excluding other causes of liver dysfunction (16). Polyhydramnios is amniotic fluid volume (AFV) ≥8 cm or amniotic fluid index (AFI) ≥25 cm, while oligohydramnios is amniotic fluid volume (AFV) ≤ 2 cm or amniotic fluid index (AFI) ≤ 5 cm. Preterm birth is defined as a delivery at gestational weeks <37 and ≥28 weeks. Fetal distress is defined as having progressive dyspnea and respiratory failure shortly after birth. Neonatal asphyxia is defined as neonate Apgar score of the first minute after birth ≤ 7. Neonatal hypoglycemia is defined as neonatal whole blood glucose value <2.2 mmol/L (40 mg/dL). Neonatal hyperbilirubinemia is defined as serum bilirubin of term infant >205 μmol/L (12 mg/dL) or serum bilirubin of preterm infant >256 μmol/L (15 mg/dL). Birth defect is defined as abnormal structure or function metabolism of embryo or fetus. Small for gestational age (SGA) is defined as a birth weight under the 10th percentile. Low birth weight infant (LBWI) is defined as birth weight <2,500 g, and macrosomia is defined as birth weight more than 4,000 g. All diagnosis were confirmed by obstetricians or specialist physician.

### Statistical Analysis

The clinical characteristics of the study population were described as the mean ± S.D. or number (%). In the subgroup analysis, women with GDM was divided into two groups according OGTT0 (<5.1 mmol/L and ≥5.1 mmol/L groups) or OGTT1 (<10 mmol/L and ≥10 mmol/L groups) or OGTT2 (<8.5 mmol/L and ≥8.5 mmol/L groups), separately. Parameters between two groups in each subgroup were further compared by *T*-test and χ^2^ test. Binary logistic regression model was used to analyze the association between OGTT results and adverse pregnancy outcomes. Adjustment was made for age, parity, IVF or AID, scarred uterus, hypothyroidism, hyperthyroidism, HBV, inflammation of vagina, history of spontaneous abortion, history of ectopic pregnancy. The data analyses were performed with SPSS 24.0 (IBM Corp, Armonk, NY, USA).

Restricted cubic spline (RCS) is piecewise cubic polynomials connected across different intervals of a continuous variable. Advanced maternal age is defined as childbearing in a woman over 35 years of age (17, 18). Women with advanced age are considered to have an increased risk of GDM and have greater risks of adverse pregnancy complications (19, 20). Therefore, we want to further explore the linear and non-linear relationship between blood glucose and adverse pregnancy outcomes in elderly (≥35 years old) and younger (<35 years old) pregnant women with GDM. To investigate non-linearity or linearity and the shapes of the associations stratified by age (<35 and ≥35 years old) between OGTT results and adverse outcomes, logistic regression models including RCS function with four knots (percentile 5, 35, 65, 95) were conducted (21). These models were adjusted for parity, IVF or AID, scarred uterus, hypothyroidism, hyperthyroidism, HBV, inflammation of vagina, history of spontaneous abortion, history of ectopic pregnancy. Odd ratios (OR) were derived from models by calculating the odds at each value of the markers and dividing this by the odds of a reference category (percentile 25 of independent variable). Probabilities for developing adverse pregnancy outcomes were also computed from the models and plotted for each of the markers. The statistical analyses were done with R version 3.5.2, using the packages rms and ggplot2.

Additional path model analysis of mediating effects was used to evaluate associations among age, OGTT and adverse outcomes in GDM patients. We used to employ this method in our previous studies to identify mediating effect among age, gene promoter methylation level and type 2 diabetes mellitus (T2DM) vascular complications (22). Because adverse outcome was binary variable, we used Bayesian analysis method to get the standardized regression coefficients a, b, c′ and c which identify relationships between variables (23) (in the model the variables are a, direct association between independent and mediator variable; b, direct association between mediator and dependent variable; c′, direct association between independent and dependent variable; c, total association between independent and dependent variables; ab, indirect effect; c′, direct effect; c, total effect; and (ab/c)^*^100%, mediating effect generated by the mediator variable in the dependent variable outcome caused by the independent variable). The standardized coefficient in the model is significant when the 95% CI did not include 0. The partial mediating model is effective with all effective standardized coefficients. The model evaluation criterion is posterior predictive *p*-value ranging from 0 to 1 with the acceptable quantity of 0.5 or close to it (24–26). The analyses were conducted by SPSS 24.0 and AMOS 21.0 (IBM Corp, Armonk, NY, USA).

## Result

### Population Characteristics of Total Women

Women with GDM had a mean age of 31.83 years old, and 24.49% of them had advanced maternal age (≥35 years old). Only 41.93% of women was primiparous. In the common disorders, HBV infection rate was relatively high (12.10%). The percentage of the history of spontaneous abortion 13.00%, which was higher than the history of ectopic pregnancy (2.81%). More than half of participation accepted cesarean section in this pregnancy (57.05%), while 10.39% give birth prematurely (Table 1).

**Table 1:** The baseline characteristics in women with GDM.

**Characteristics**	**Women with GDM (*n* = 6861)**
**Mother**	
Age (years)	31.83 ± 5.00
<35 (*n*, %)	5,181 (75.51%)
≥35 (*n*, %)	1,680 (24.49%)
Parity	
0 (*n*, %)	2,877 (41.93%)
1 (*n*, %)	3,475 (50.65%)
≥2 (*n*, %)	509 (7.42%)
IVF or AID (*n*, %)	530 (7.72%)
Scarred uterus (*n*, %)	1,927 (28.09%)
Hypothyroidism (*n*, %)	257 (3.75%)
Hyperthyroidism (*n*, %)	57 (0.83%)
HDP (*n*, %)	337 (4.91%)
HBV (*n*, %)	830 (12.10%)
Inflammation of vagina (*n*, %)	271 (3.95%)
History of spontaneous abortion (*n*, %)	892 (13.00%)
History of ectopic pregnancy (*n*, %)	193 (2.81%)
Cesarean section (*n*, %)	3,914 (57.05%)
ICP (*n*, %)	51 (0.74%)
Polyhydramnios (*n*, %)	99 (1.44%)
Oligohydramnios (*n*, %)	476 (6.84%)
PROM (*n*, %)	1,238 (18.04%)
**Newborn infant or fetus**	
Male infant gender (*n*, %)	3,731 (54.38%)
Preterm neonates (*n*, %)	713 (10.39%)
Fetal distress (*n*, %)	504 (7.35%)
Neonatal asphyxia (*n*, %)	88 (1.28%)
Neonatal hypoglycemia (*n*, %)	78 (1.14%)
Hyperbilirubinemia (*n*, %)	603 (8.79%)
Birth defect (*n*, %)	270 (3.94%)
SGA (*n*, %)	177 (2.58%)
LBWI (*n*, %)	552 (8.05%)
Macrosomia (*n*, %)	530 (7.72%)

*GDM, gestational diabetes mellitus; IVF, in vitro fertilization; AID, artificial insemination; HDP, hypertensive disorders of pregnancy; HBV, Hepatitis B Virus; ICP, intrahepatic cholestasis of pregnancy; PROM, premature rupture of membranes; SGA, small for gestational age; LBWI, low birth weight infants*.

### Population Characteristic of GDM Subgroups

In OGTT0 subgroups, compared with OGTT0 <5.1 group, OGTT0 ≥5.1 group had older age (32.05 vs. 31.76 years), more parity and higher percentage of scarred uterus HDP, cesarean section, preterm neonates, neonates hyperbilirubinemia, and macrosomia, but lower percentage of IVF or AID and HBV. In OGTT1 subgroups, OGTT1 ≥10 group was older than OGTT1 <10 group (32.04 vs. 31.54 years) and had higher percentage of scarred uterus. In addition, the incidence of HDP, cesarean section, preterm neonates and neonatal hyperbilirubinemia were substantially increased in OGTT1 ≥10 group. In OGTT2 subgroups, all of adverse outcomes did not significantly differ between OGTT2 ≥8.5 group and OGTT2 <8.5 group (*P* > 0.05). Maternal age was positive correlated with higher OGTT2 glucose level (Table 2).

**Table 2:** Baseline characteristics according to OGTT in women with GDM.

**Characteristics**	**OGTT0 <5.1** **(*n* = 5,195)**	**OGTT0 ≥5.1** **(*n* = 1,666)**	***P*-value**	**OGTT1 <10** **(*n* = 2,822)**	**OGTT0 ≥10** **(*n* = 4,039)**	***P*-value**	**OGTT2 <8.5** **(*n* = 2,397)**	**OGTT2 ≥8.5** **(*n* = 4,464)**	***P*-value**
**Mother**									
Age (years)	31.76 ± 4.95	32.05 ± 5.14	0.044^*^	31.54 ± 4.99	32.04 ± 5.00	<0.001^**^	30.78 ± 4.85	32.40 ± 4.99	<0.001^**^
<35 (*n*, %)	3,947 (75.98%)	1,234 (74.07%)		2,190 (77.60%)	2,991 (74.05%)		1,962 (81.85%)	3,219 (72.11%)	
≥35 (*n*, %)	1,248 (24.02%)	432 (25.93%)	0.115	632 (22.40%)	1,048 (25.95%)	0.001^*^	435 (18.15%)	1,245 (27.89%)	<0.001^**^
Parity									
0 (*n*, %)	2,277 (43.83%)	600 (36.01%)		1,195 (42.35%)	1,682 (41.64%)		1,066 (44.47%)	1,811 (40.57%)	
1 (*n*, %)	2,589 (49.84%)	886 (53.18%)		1,422 (50.39%)	2,053 (50.83%)		1,145 (47.77%)	2,330 (52.20%)	
≥2 (*n*, %)	329 (6.33%)	180 (10.80%)	<0.001^**^	205 (7.26%)	302 (7.53%)	0.814	186 (7.76%)	323 (7.24%)	0.002^*^
IVF or AID (*n*, %)	426 (8.20%)	104 (6.24%)	0.009^*^	204 (7.23%)	326 (8.07%)	0.198	147 (6.13%)	383 (8.58%)	<0.001^**^
Scarred uterus (*n*, %)	1,368 (26.33%)	559 (33.55%)	<0.001^**^	706 (25.02%)	1,221 (30.23%)	<0.001^**^	634 (26.45%)	1,293 (28.97%)	0.027^*^
Hypothyroidism (*n*, %)	198 (3.81%)	59 (3.54%)	0.614	103 (3.65%)	154 (3.81%)	0.727	82 (3.42%)	175 (3.92%)	0.299
Hyperthyroidism (*n*, %)	44 (0.85%)	13 (0.78%)	0.794	23 (0.82%)	34 (0.84%)	0.904	15 (0.626%)	42 (0.941%)	0.170
HDP (*n*, %)	216 (4.16%)	121 (7.26%)	<0.001^**^	106 (3.76%)	231 (5.72%)	<0.001^**^	104 (4.34%)	233 (5.22%)	0.108
HBV (*n*, %)	657 (12.65%)	173 (10.38%)	0.014^*^	339 (12.01%)	491 (12.16%)	0.857	265 (11.06%)	565 (12.66%)	0.052
Inflammation of vagina (*n*, %)	197 (3.79%)	74 (4.44%)	0.236	118 (4.18%)	153 (3.79%)	0.410	112 (4.67%)	159 (3.56%)	0.024^*^
History of spontaneous abortion (*n*, %)	685 (13.19%)	207 (12.42%)	0.422	360 (12.76%)	532 (13.17%)	0.615	276 (11.51%)	616 (13.80%)	0.007^*^
History of ectopic pregnancy (*n*, %)	137 (2.64%)	56 (3.36%)	0.120	77 (2.73%)	116 (2.87%)	0.724	55 (2.29%)	138 (3.09%)	0.057
Cesarean section (*n*, %)	2,875 (55.34%)	1,039 (62.36%)	<0.001^**^	1,521 (53.90%)	2,393 (59.24%)	<0.001^**^	1,295 (54.02%)	2,619 (58.67%)	<0.001^**^
ICP (*n*, %)	34 (0.65%)	17 (1.02%)	0.130	17 (0.60%)	34 (0.84%)	0.256	18 (0.75%)	33 (0.74%)	0.957
Polyhydramnios (*n*, %)	70 (1.35%)	29 (1.74%)	0.242	53 (1.88%)	46 (1.14%)	0.012^*^	34 (1.42%)	65 (1.46%)	0.901
Oligohydramnios (*n*, %)	369 (7.10%)	107 (6.42%)	0.342	184 (6.25%)	292 (7.23%)	0.255	170 (7.09%)	306 (6.85%)	0.712
PROM (*n*, %)	943 (18.15%)	295 (17.71%)	0.681	525 (18.60%)	713 (17.65%)	0.314	433 (18.06%)	805 (18.03%)	0.975
**Newborn infant or fetus**									
Male infant gender (*n*, %)	2,809 (54.07%)	922 (55.34%)	0.365	1,478 (52.34%)	2,253 (55.78%)	0.005^*^	1,345 (56.11%)	2,386 (53.45%)	0.035^*^
Preterm neonates (*n*, %)	510 (9.82%)	203 (12.18%)	0.006^*^	268 (9.50%)	445 (11.02%)	0.042^*^	232 (9.68%)	481 (10.78%)	0.156
Fetal distress (*n*, %)	338 (7.47%)	116 (6.96%)	0.491	196 (6.95%)	308 (7.63%)	0.288	187 (7.80%)	317 (7.10%)	0.289
Neonatal asphyxia (*n*, %)	63 (1.21%)	25 (1.50%)	0.364	45 (1.59%)	43 (1.06%)	0.055	28 (1.17%)	60 (1.34%)	0.537
Neonatal hypoglycemia (*n*, %)	52 (1.00%)	26 (1.56%)	0.061	27 (0.96%)	51 (1.26%)	0.240	24 (1.00%)	54 (1.21%)	0.438
Hyperbilirubinemia (*n*, %)	430 (8.28%)	173 (10.38%)	0.008^*^	224 (7.94%)	379 (9.38%)	0.037^*^	197 (8.22%)	406 (9.09%)	0.222
Birth defect (*n*, %)	195 (3.75%)	75 (4.50%)	0.172	118 (4.18%)	152 (3.76%)	0.382	92 (3.83%)	178 (3.99%)	0.760
SGA (*n*, %)	137 (2.64%)	40 (2.40%)	0.597	80 (2.83%)	97 (2.40%)	0.265	54 (2.25%)	123 (2.78%)	0.211
LBWI (*n*, %)	402 (7.34%)	150 (9.00%)	0.098	226 (8.01%)	326 (8.07%)	0.925	184 (7.68%)	368 (8.24%)	0.410
Macrosomia (*n*, %)	379 (7.30%)	151 (9.06%)	0.019^*^	203 (7.19%)	327 (8.10%)	0.168	167 (6.97%)	363 (8.13%)	0.085

IVF, in vitro fertilization; AID, artificial insemination; HDP, hypertensive disorders of pregnancy; HBV, Hepatitis B Virus; ICP, intrahepatic cholestasis of pregnancy; PROM, premature rupture of membranes; SGA, small for gestational age; LBWI, low birth weight infants; NBWI, normal birth weight infants.

** P < 0.001,

* P < 0.05.

*Bold values indicate statistically significant (P < 0.05)*.

### Association Between OGTT and Adverse Pregnancy Outcomes in Multiple Logistic Regression

In logistic regression with adjustment, GDM patients with OGTT0 ≥5.1 had 1.891 times higher risk (Adj *P* < 0.001) of developing HDP than those with OGTT0 <5.1. A higher OGTT0 level (OGTT0 ≥5.1) and the risk of preterm neonates, neonatal hyperbilirubinemia and macrosomia were also observed, with 1.284, 1.285, and 1.302 times increases in susceptibility (Adj *P* = 0.050, Adj *P* = 0.090, Adj *P* = 0.010), respectively. GDM patients with OGTT1 ≥10 had only found to exhibited 1.473-fold (Adj *P* = 0.001) increasing risk of developing HDP compared to those with OGTT1 <10. No adverse outcome was identified to associate with higher OGTT2 (≥8.5 mmol/L) (Adj *P* > 0.05) (Table 3).

**Table 3:** Risk of adverse pregnancy outcomes according to OGTT in women with GDM.

**Outcome**		**OGTT0 <5.1 OR (95% CI)**	**OGTT0 ≥5.1** **OR (95% CI)**	***P*-value**	**OGTT1 <10** **OR (95% CI)**	**OGTT0 ≥10 OR (95% CI)**	***P-*value**	**OGTT2 <8.5 OR (95% CI)**	**OGTT2 ≥8.5** **OR (95% CI)**	***P*-value**
**Mother**										
HDP	Unadj	1.00	**1.805 (1.435–2.273)**	<0.001^**^	1.00	**1.554 (1.229–1.966)**	<0.001^**^	1.00	1.214 (0.958**–**1.538)	0.108
	Adj	1.00	**1.819 (1.441–2.298)**	<0.001^**^	1.00	**1.473 (1.162–1.867)**	0.001^*^	1.00	1.047 (0.822**–**1.334)	0.708
ICP	Unadj	1.00	1.565 (0.872**–**2.808)	0.133	1.00	1.404 (0.781**–**2.512)	0.258	1.00	0.984 (0.553**–**1.752)	0.957
	Adj	1.00	1.666 (0.925**–**2.999)	0.089	1.00	1.401 (0.779**–**2.518)	0.260	1.00	0.962 (0.536**–**1.728)	0.897
Polyhydramnios	Unadj	1.00	1.297 (0.838**–**2.007)	0.243	1.00	0.602 (0.404**–**1.012)	0.058	1.00	1.027 (0.676**–**1.560)	0.901
	Adj	1.00	1.254 (0.807**–**1.947)	0.314	1.00	0.663 (0.434**–**1.014)	0.059	1.00	0.908 (0.594**–**1.390)	0.658
Oligohydramnios	Unadj	1.00	0.898 (0.718**–**1.121)	0.342	1.00	1.117 (0.923**–**1.353)	0.255	1.00	0.964 (0.794**–**1.171)	0.712
	Adj	1.00	0.946 (0.756**–**1.185)	0.631	1.00	1.138 (0.939**–**0.897)	0.186	1.00	0.976 (0.800**–**1.189)	0.807
PROM	Unadj	1.00	0.970 (0.840**–**1.121)	0.681	1.00	0.938 (0.828**–**1.062)	0.314	1.00	0.998 (0.877**–**1.135)	0.975
	Adj	1.00	1.032 (0.888**–**1.199)	0.681	1.00	0.967 (0.850**–**1.100)	0.606	1.00	1.023 (0.894**–**1.171)	0.740
**Newborn infant or fetus**										
Preterm neonates	Unadj	1.00	**1.275 (1.072–1.515)**	0.006^*^	1.00	**1.180 (1.006–1.384)**	0.042^*^	1.00	1.127 (0.955**–**1.329)	0.156
	Adj	1.00	**1.284 (1.078–1.529)**	0.005^*^	1.00	1.172 (0.998**–**1.146)	0.053	1.00	1.090 (0.921–1.290)	0.314
Fetal distress	Unadj	1.00	0.927 (0.748–1.150)	0.491	1.00	1.106 (0.918–1.332)	0.288	1.00	0.903 (0.749–1.090)	0.289
	Adj	1.00	1.011 (0.814–1.257)	0.918	1.00	1.135 (0.941–1.369)	0.187	1.00	0.917 (0.757–1.111)	0.374
Neonatal asphyxia	Unadj	1.00	1.241 (0.778–1.979)	0.364	1.00	0.664 (0.436–1.011)	0.057	1.00	1.153 (0.734–1.810)	0.537
	Adj	1.00	1.252 (0.783–2.003)	0.349	1.00	0.664 (0.435–1.013)	0.058	1.00	1.193 (0.755–1.884)	0.450
Neonatal hypoglycemia	Unadj	1.00	1.568 (0.976–2.519)	0.063	1.00	1.324 (0.828–2.116)	0.241	1.00	1.211 (0.747–1.963)	0.438
	Adj	1.00	1.542 (0.956–2.488)	0.076	1.00	1.268 (0.792–2.031)	0.323	1.00	1.109 (0.679–1.811)	0.679
Hyperbilirubinemia	Unadj	1.00	**1.284 (1.066**–**1.546)**	0.008^*^	1.00	**1.201 (1.011**–**1.427)**	0.038^*^	1.00	1.117 (0.935–1.335)	0.222
	Adj	1.00	**1.285 (1.065**–**1.550)**	0.009^*^	1.00	1.156 (1.972–1.375)	0.102	1.00	1.064 (0.888–1.275)	0.501
Birth defect	Unadj	1.00	1.208 (0.920–1.587)	0.173	1.00	0.896 (0.701–1.146)	0.382	1.00	1.041 (0.805–1.345)	0.760
	Adj	1.00	1.185 (0.901–1.558)	0.226	1.00	0.883 (0.690–1.130)	0.323	1.00	1.007 (0.776–1.306)	0.960
SGA	Unadj	1.00	0.908 (0.636–1.297)	0.597	1.00	0.843 (0.625–1.138)	0.266	1.00	1.229 (0.889–1.700)	0.211
	Adj	1.00	0.937 (0.654–1.340)	0.720	1.00	0.841 (0.622–1.136)	0.259	1.00	1.231 (0.887–1.710)	0.215
LBWI	Unadj	1.00	1.180 (0.970–1.435)	0.099	1.00	1.009 (0.845–1.204)	0.925	1.00	1.081 (0.899–1.299)	0.410
	Adj	1.00	1.187 (0.974–1.448)	0.090	1.00	1.004 (0.840–1.199)	0.967	1.00	1.086 (0.900–1.310)	0.390
Macrosomia	Unadj	1.00	**1.267 (1.040**–**1.543)**	0.019^*^	1.00	1.137 (0.947–1.364)	0.168	1.00	1.182 (0.977–1.430)	0.085
	Adj	1.00	**1.302 (1.067**–**1.590)**	0.010^*^	1.00	1.154 (0.961–1.387)	0.126	1.00	1.200 (0.988–1.457)	0.066

IVF, in vitro fertilization; AID, artificial insemination; HDP, hypertensive disorders of pregnancy; ICP, intrahepatic cholestasis of pregnancy; PROM, premature rupture of membranes; SGA, small for gestational age; LBWI, low birth weight infants; NBWI, normal birth weight infants.

Adj, adjusted for age, parity, IVF or AID, scarred uterus, hypothyroidism, hyperthyroidism, HBV, inflammation of vagina, history of spontaneous abortion, history of ectopic pregnancy.

** P < 0.001,

* P < 0.05.

*Bold values indicate statistically significant (P < 0.05)*.

### Association Between OGTT and Adverse Pregnancy Outcomes in RCS Nested Logistic Regression

**Figure 1:**
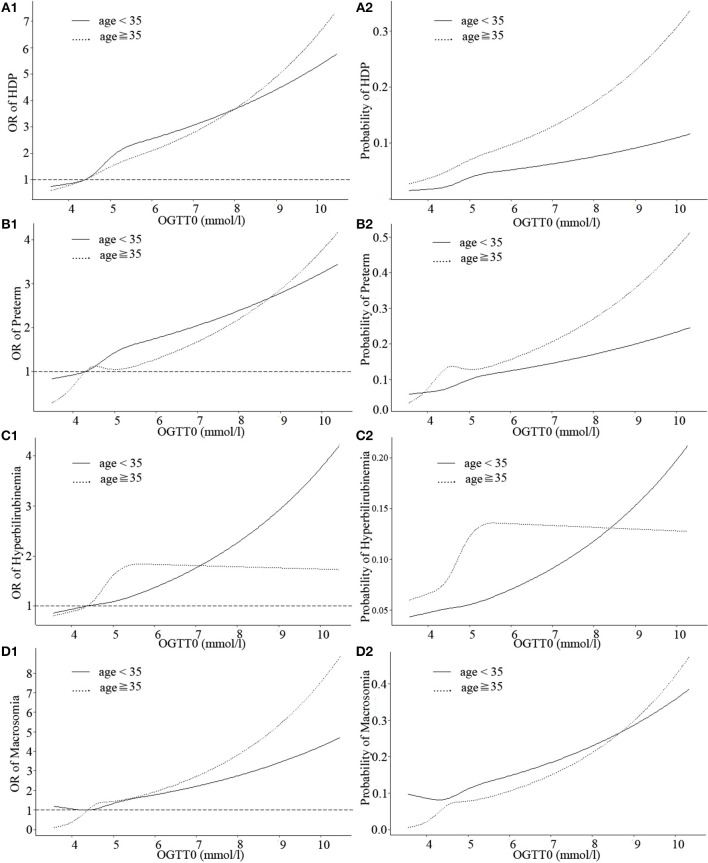
Curves of adjusted OR and absolute risks stratified by age derived from logistic regression models including restricted cubic splines (RCS) to evaluate the associations of adverse pregnancy outcomes of gestational diabetes mellitus (GDM) with OGTT0. The reference OGTT0 for these plot (with OR fixed as 1) was 25% of OGTT0 (4.3 mmol/l). **(A1)** OR of HDP, hypertensive disorders of pregnancy; **(A2)** Probability of HDP, hypertensive disorders of pregnancy; **(B1)** OR of preterm; **(B2)** Probability of preterm; **(C1)** OR of hyperbilirubinemia; **(C2)** Probability of hyperbilirubinemia; **(D1)** OR of macrosomia; **(D2)** Probability of macrosomia. Adjustment was made for parity, IVF or AID, scarred uterus, hypothyroidism, hyperthyroidism, HBV, inflammation of vagina, history of spontaneous abortion, history of ectopic pregnancy.

Figure 1 showed the estimated size and shape of the associations stratified by age of the OGTT0 glucose level with HDP, preterm, neonatal hyperbilirubinemia, and macrosomia incidence, all allowing for linearity (Table S1). Increases in HDP and preterm risk with OGTT0 in GDM patients over age 35 were larger than those under age 35. Association between OGTT0 and neonatal hyperbilirubinemia was more marked in GDM patients under age 35 than over age 35: in women under age 35, higher OGTT0 was associated with substantially increased risk, whereas in women over age 35 more modest decrease in risk if OGTT0 was <5.3 mmol/l. The probability of neonatal hyperbilirubinemia in GDM patients under age 35 was higher than those over age 35 when OGTT0 was more than 8.3 mmol/l, while macrosomia of women under age 35 was lower than over age 35 if OGTT0 was <8.6 mmol/l.

**Figure 2:**
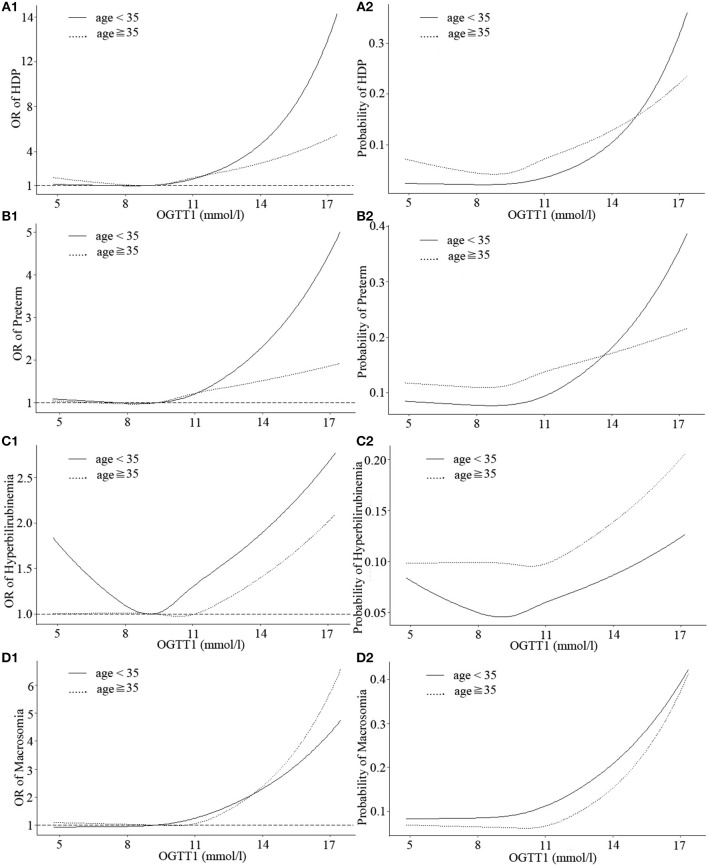
Curves of adjusted OR and absolute risks stratified by age derived from logistic regression models including restricted cubic splines (RCS) to evaluate the associations of adverse pregnancy outcomes of gestational diabetes mellitus (GDM) with OGTT1. The reference OGTT1 for these plot (with OR fixed as 1) was 25% of OGTT1 (9.28 mmol/l). **(A1)** OR of HDP, hypertensive disorders of pregnancy; **(A2)** Probability of HDP, hypertensive disorders of pregnancy; **(B1)** OR of preterm; **(B2)** Probability of preterm; **(C1)** OR of hyperbilirubinemia; **(C2)** Probability of hyperbilirubinemia; **(D1)** OR of macrosomia; **(D2)** Probability of macrosomia. Adjustment was made for parity, IVF or AID, scarred uterus, hypothyroidism, hyperthyroidism, HBV, inflammation of vagina, history of spontaneous abortion, history of ectopic pregnancy.

**Figure 3:**
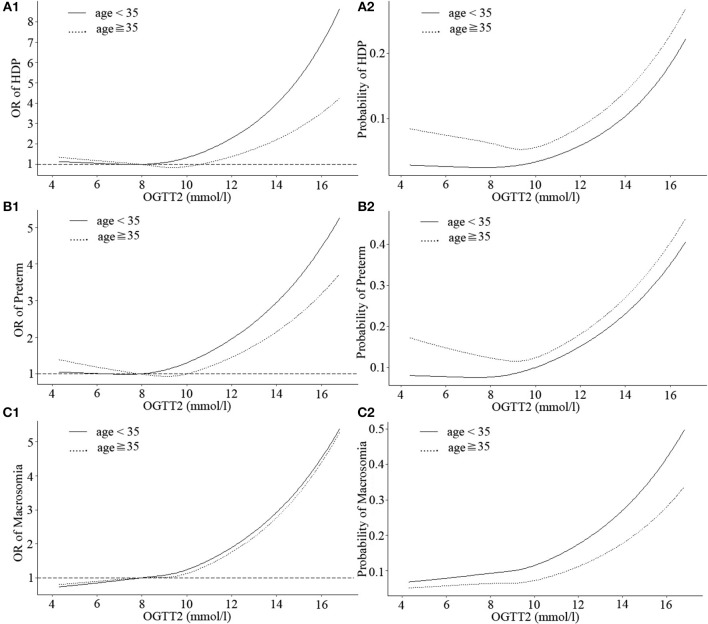
Curves of adjusted OR and absolute risks stratified by age derived from logistic regression models including restricted cubic splines (RCS) to evaluate the associations of adverse pregnancy outcomes of gestational diabetes mellitus (GDM) with OGTT2. The reference OGTT2 for these plot (with OR fixed as 1) was 25% of OGTT2 (7.92 mmol/l). **(A1)** OR of HDP, hypertensive disorders of pregnancy; **(A2)** Probability of HDP, hypertensive disorders of pregnancy; **(B1)** OR of preterm; **(B2)** Probability of preterm; **(C1)** OR of macrosomia; **(C2)** Probability of macrosomia. Adjustment was made for parity, IVF or AID, scarred uterus, hypothyroidism, hyperthyroidism, HBV, inflammation of vagina, history of spontaneous abortion, history of ectopic pregnancy.

The shapes of the associations between OGTT1 and HDP, preterm, neonatal hyperbilirubinemia, and macrosomia deviated from linear, although non-linearity was most evident in HDP, preterm, and neonatal hyperbilirubinemia of GDM patients under age 35 (non-linear *P* = 0.037, 0.049, and 0.039, respectively). In GDM patients under age 35, OGTT1 was negatively related to neonatal hyperbilirubinemia at the lower end of the of OGTT1 range (<9.5 mmol/l), whereas above this value, risk of neonatal hyperbilirubinemia increased markedly with increasing OGTT1 (Figure 2, Table S1). A similar nadir point pattern was noted for HDP and preterm with OGTT2 in women more than 35 years old (with two risk nadir points both at OGTT2 9.5 mmol/l) (Figure 3).

Three continuous values of OGTT had differences in the strengths of associations with HDP, preterm, neonatal hyperbilirubinemia and macrosomia among GDM patients, but as a whole, the probability of obtaining these outcomes was at a similar range from 0.2 to 0.5. Moreover, when the three OGTT values of GDM patients were >5.1, 10, or 8.5 mmol/l, respectively, incidence of HDP, preterm, neonatal hyperbilirubinemia, and macrosomia would sharply increase.

### OGTT Had Mediating Effects Between Age and Adverse Pregnancy Outcomes

**Figure 4:**
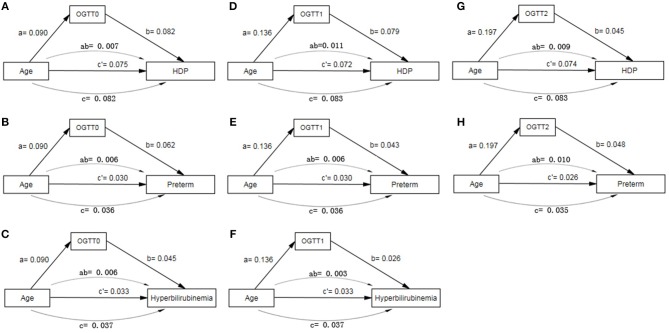
Path model analysis with standardized coefficients of age, OGTT and adverse pregnancy outcomes of GDM. **(A)** Path model of age, OGTT0 and HDP; **(B)** Path model of age, OGTT0 and Preterm; **(C)** Path model of age, OGTT0 and hyperbilirubinemia; **(D)** Path model of age, OGTT1 and HDP; **(E)** Path model of age, OGTT1 and Preterm; **(F)** Path model of age, OGTT1 and hyperbilirubinemia; **(G)** Path model of age, OGTT2 and HDP; **(H)** Path model of age, OGTT2 and Preterm. AOMS Bayesian analysis method was used in path model analysis.

In path analysis, OGTT0 and OGTT1 were both found to have partially mediating effects between age and HDP, preterm and neonatal hyperbilirubinemia, and OGTT2 had the same effect between age and HDP and preterm (Figure 4, Table S2). The mediating effect of OGTT in all 11 path models was about 8–30%. That is to say, the older GDM patients are, the greater risk of these adverse outcomes, and age could increase the risk of the outcomes by higher OGTT glucose level at the same time.

## Discussion

The study here showed the associations between higher FPG (OGTT0 ≥5.1) and HDP, preterm, neonatal hyperbilirubinemia, and macrosomia among women with GDM, with similar relationship between higher 1-hour PG (OGTT1 ≥10) and HDP. We found that OGTT0 was linearly associated with incidence of HDP, preterm, neonatal hyperbilirubinemia and macrosomia in both age groups (<35 and ≥ 35). Non-linear associations of OGTT1 with incidence of HDP, preterm, and neonatal hyperbilirubinemia were detected in younger patients. OGTT2 was linearly associated with HDP, preterm, and macrosomia in both age groups except for HDP in the older age group. OGTT results partially mediated the effect between age and HDP, preterm, and neonatal hyperbilirubinemia in GDM.

GDM could lead to various adverse pregnancy outcomes, and different hyperglycemia characteristics of three values in OGTT might result in different degree of pregnancy complications risks, which was consistent with Metzger et al. (5). We found that GDM patients with higher OGTT0 (OGTT0 ≥5.1) had approximately 1.5 times higher risk of developing HDP, preterm, neonatal hyperbilirubinemia, or macrosomia than those women with lower OGTT0 (OGTT0 <5.1). However, little difference of adverse pregnancy outcomes of GDM was identified to be related to OGTT1 or OGTT2. Not all three abnormal OGTT values resulted in the same adverse complications or the same risk of specific adverse outcomes. Higher FPG was also reported to be associated with macrosomia, LGA and neonatal adiposity (27, 28). HAPO investigators showed that a woman's response to glucose was at least partially correlated with her FPG levels (5). Associations between post-load glucose and primary outcomes were weaker than for fasting glucose (29). Therefore, fasting hyperglycemia closely linked to glucose metabolic abnormality might be more prone to causing adverse perinatal outcomes, and FPG might be the strongest predictor, which warrants a tailored management strategy for GDM. Since there has no clear and uniform guideline for treating GDM with prescription drugs in mainland China nowadays, all participants didn't treated with oral hypoglycemic drugs in pregnancy. Further studies should be focused on the use of hypoglycemic medication for Chinese pregnant women, and relevant guidelines, especially for regulating PFG to lower risk of adverse outcomes, are urgently needed.

Information on the non-linear associations between maternal glucose and incidence of GDM or adverse pregnancy outcomes of GDM patients is limited. Each value of OGTT was said to be a significant predictor of primary cesarean section when the glucose values were analyzed as continuous variables (5). A meta-analysis reported that there was a consistent graded linear association between fasting and post-load glucose and perinatal outcomes including cesarean section, macrosomia, and shoulder dystocia in non-GDM women, with no clear threshold (30). Our current finding was linear relationships between fasting and post-load glucose and macrosomia among GDM patients in both two age groups (age <35 and ≥35 years), which was consistent with the meta-analysis in some way. It was suggested that FPG and HbA1c had curvilinear associations with T2DM, and rose more steeply at higher values (8, 9). In our results, three continuous OGTT values had different strengths in associations with HDP, preterm, neonatal hyperbilirubinemia, and macrosomia among GDM patients, but as a whole, the probability of obtaining these outcomes was at a similar range from 0.2 to 0.5. When the three OGTT values of GDM patients were >5.1, 10, or 8.5 mmol/L, respectively, incidence of HDP, preterm, neonatal hyperbilirubinemia, and macrosomia would sharply increase. Moreover, non-linear relationships were found between OGTT1 and HDP, preterm as well as neonatal hyperbilirubinemia in younger GDM patients. Advanced maternal age was identified as one of high risk factors of GDM (1), so it might be the risk factor for gestational complications among GDM patients. In addition, the potential explanation for differences in the size and shape of associations is diversity in the intra-individual variation between glucose markers and diversity in age variation of GDM patients. Several studies have demonstrated various degrees of intra-individual variation for FPG and post-load glucose (31, 32). Thus, both non-linearity and linearity of correlations should be taken into account when extrapolating OGTT data on complications risks for GDM patients. Moreover, once a pregnant woman is diagnosed with GDM, the OGTT values should be taken seriously to estimate the potential possibility for complications, and take some precautions in advance. As one composite score model of adverse maternal-birth outcomes was reported to evaluate risk assessment and glucose controlling benefits for individual pregnant women (33), a similar composite score model related OGTT is needed for assessing individually potential risk of complications among GDM patients. The OGTT values could also be useful when it comes to risk stratification and decisions associated with delivery and neonatal surveillance.

We found that fasting and post-load glucose had partially mediating effects on age and adverse outcomes including HDP, preterm, and neonatal hyperbilirubinemia, which indicated that not only could older age directly promote the risk of the complications, but it also could indirectly increase the risks via higher glucose. At present, few research has explored the mediating relationship among these three factors. The correlations between each two elements were reported. Older maternal age was closely correlated to incidence of GDM, and advanced age had impact on pregnancy outcomes (5). A number of studies have demonstrated the associations between maternal glucose and perinatal outcomes, such as preterm (5, 27). Associations with pre-eclampsia and glucose were reported, with OR ranged from 1.21 to 1.28 for each one S.D. increase in each glucose measure of OGTT, while preterm and hyperbilirubinemia were also related to postprandial glucose (34). Thus, age and glucose might be the vital risk factors for these adverse outcomes, and OGTT might be an important mediating element. However, further mechanistic investigation should be performed.

The strength of our study is the use of RCS nested logistic regression model exploring non-linear or linear relationships between OGTT and adverse outcomes among GDM patients. In addition, the path analysis could add more information among maternal age, glucose, and complications, which were useful for GDM management. However, several limitations in the study should be mentioned. First, we did not include information on pre-gestational BMI, maternal BMI and living habits, making it impossible to evaluate the potential effect of these variables. Second, the absence of the nutritional status of patients could affect fetal health and other perinatal outcomes. Finally, although the direct or indirect glucose intervention for all GDM patients in our hospital would be conducted, which was likely to bias the results, the study could not provide an estimated measure of it because of the absent data. We would explore the potential association in the further study.

In conclusion, higher FPG was more strongly associated with incidence of adverse pregnancy outcomes among GDM patients than post-load plasma glucose. Both linearity and Non-linearity of associations between glucose and complications should be taken into account. It is important to strengthen GDM management in the reproductive-aged women. These findings have potential implications for accurately estimating the risk of adverse pregnant outcomes on the basis of OGTT values, and FPG should be attached great importance.

## Data Availability Statement

The datasets generated for this study are available from the corresponding author Zhengping Liu ( liuzphlk81@outlook.com) on reasonable request.

## Ethics Statement

This study was carried out in accordance with the Ethics Committee of Southern Medical University Affiliated Maternal & Child Health Hospital of Foshan city with written informed consent from all subjects. All subjects gave written informed consent in accordance with the Declaration of Helsinki. The protocol was approved by the Ethics Committee of Southern Medical University Affiliated Maternal & Child Health Hospital of Foshan city.

## Author Contributions

ZZ, DS, XG, and ZL conceived and designed the study. ZZ, GC, DF, PL, SW, JR, DL, HM, SY, HZ, XS, YW, and XL carried out data collection. ZZ carried out the statistical analyses and drafted the manuscript.

### Conflict of Interest

The authors declare that the research was conducted in the absence of any commercial or financial relationships that could be construed as a potential conflict of interest.
