# Supplementary figures and images for: Size and Shape of Associations of OGTT as Well as Mediating Effects on Adverse Pregnancy Outcomes Among Women With Gestational Diabetes Mellitus: Population-Based Study From Southern Han Chinese
Source: Front Endocrinol (Lausanne). 2020 Mar 17;11:135. doi: 10.3389/fendo.2020.00135 (PMC7092640; doi:10.3389/fendo.2020.00135)

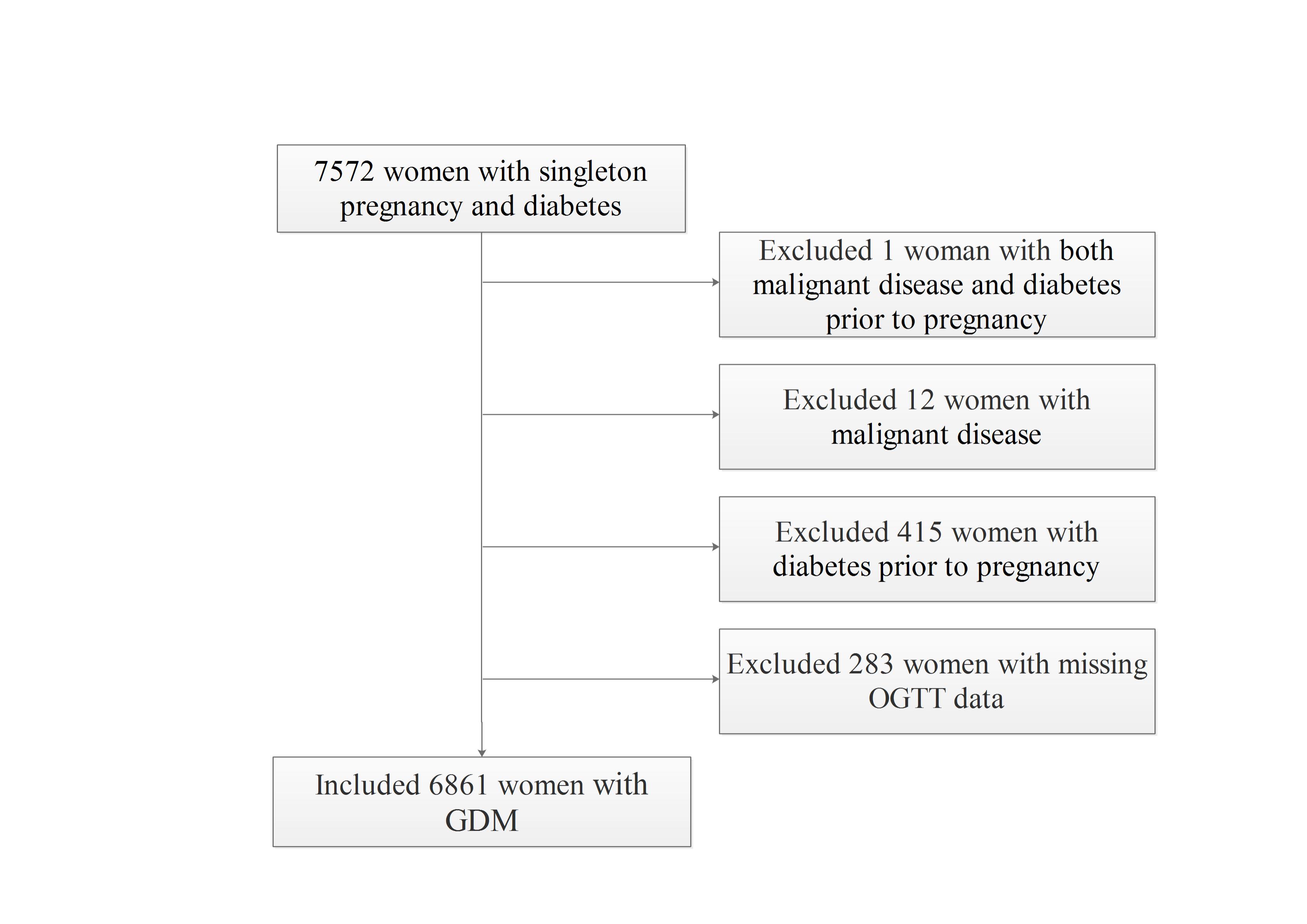

Supplement: Supplementary file 1 [file Image_1.jpeg]
