# Supplementary material for: Size and Shape of Associations of OGTT as Well as Mediating Effects on Adverse Pregnancy Outcomes Among Women With Gestational Diabetes Mellitus: Population-Based Study From Southern Han Chinese
Source: Front Endocrinol (Lausanne). 2020 Mar 17;11:135. doi: 10.3389/fendo.2020.00135 (PMC7092640; doi:10.3389/fendo.2020.00135)
Supplement: Supplementary file 2 [file Data_Sheet_1.docx]

**Supplementary material**

**Figure legend**

Figure S1 The follow charts of study population.

Table S1 The statistic information of logistic regression models with RCS functions

| Model | Total χ^2^ statistics | Total P-value | Non-linear χ^2^ statistics | Non-linear P-value | Independent variable OGTT χ^2^ statistics | Independent variable OGTT P-value |
| --- | --- | --- | --- | --- | --- | --- |
| Outcome-OGTT0 |  |  |  |  |  |  |
| HDP-OGTT0  Age <35 | 52.84 | <0.001** | 5.60 | 0.061 | 25.91 | <0.001** |
| HDP-OGTT0  Age ≧35 | 37.92 | <0.001** | 1.25 | 0.536 | 17.16 | <0.001** |
| Preterm-OGTT0  Age <35 | 58.37 | <0.001** | 2.50 | 0.286 | 21.12 | <0.001** |
| Preterm-OGTT0  Age ≧35 | 40.08 | <0.001** | 3.19 | 0.203 | 10.88 | 0.012* |
| Hyperbilirubinemia-OGTT0 Age <35 | 42.52 | <0.001** | 0.25 | 0.883 | 7.93 | 0.047* |
| Hyperbilirubinemia-OGTT0 Age≧35 | 23.46 | 0.024* | 5.64 | 0.060 | 11.34 | 0.001* |
| Macrosomia-  OGTT0 Age <35 | 79.27 | <0.001** | 2.01 | 0.365 | 17.44 | 0.001* |
| Macrosomia-  OGTT0 Age≧35 | 32.52 | <0.001** | 3.74 | 0.154 | 18.67 | <0.001** |
| Outcome-OGTT1 |  |  |  |  |  |  |
| HDP-OGTT1  Age <35 | 69.22 | <0.001** | 6.60 | 0.037* | 42.92 | <0.001** |
| HDP-OGTT1  Age ≧35 | 37.62 | <0.001** | 2.52 | 0.283 | 16.62 | <0.001** |
| Preterm-OGTT1 Age <35 | 56.24 | <0.001** | 6.13 | 0.047* | 19.22 | <0.001** |
| Preterm-OGTT1 Age ≧35 | 32.85 | 0.001** | 0.35 | 0.841 | 3.40 | 0.334 |
| Hyperbilirubinemia-OGTT1 Age <35 | 44.04 | <0.001** | 6.47 | 0.039* | 9.13 | 0.028* |
| Hyperbilirubinemia-OGTT0 Age≧35 | 14.68 | 0.260 | 1.10 | 0.577 | 2.35 | 0.503 |
| Macrosomia-  OGTT1 Age <35 | 80.85 | <0.001** | 3.08 | 0.215 | 18.41 | <0.001** |
| Macrosomia-  OGTT1 Age ≧35 | 28.69 | 0.004* | 4.49 | 0.106 | 14.65 | 0.002* |
| Outcome-OGTT2 |  |  |  |  |  |  |
| HDP-OGTT2  Age <35 | 47.42 | <0.001** | 4.91 | 0.086 | 20.22 | <0.001** |
| HDP-OGTT2  Age ≧35 | 32.34 | 0.001* | 6.74 | 0.034* | 11.19 | 0.011* |
| Preterm-OGTT2  Age <35 | 57.65 | <0.001** | 4.47 | 0.107 | 20.38 | <0.001** |
| Preterm-OGTT2 Age ≧35 | 38.80 | <0.001** | 5.61 | 0.060 | 10.00 | 0.019 |
| Macrosomia-  OGTT2 Age <35 | 81.90 | <0.001** | 2.09 | 0.351 | 19.70 | <0.001 |
| Macrosomia-  OGTT2 Age ≧35 | 25.95 | 0.011* | 1.73 | 0.420 | 11.60 | 0.009* |

**P<0.001，*P<0.05.

Table S2 The standardized regression coefficients and model evaluation value for path model analysis of mediating effects

| Model | a  (95%CI) | b  (95%CI) | c’  (95%CI)  (direct effect) | c  (95%CI)  (total effect) | ab  (95%CI)  (indirect effect) | (ab/c)*100%  (mediating effect) | Posterior predictive P-value |
| --- | --- | --- | --- | --- | --- | --- | --- |
| Age-OGTT0-HDP | 0.090 (0.066-0.113) | 0.082 (0.058-0.105) | 0.075 (0.052-0.099) | 0.082 (0.059-0.106) | 0.007 (0.005-0.010) | 8.54% | 0.5 |
| Age-OGTT0-Preterm | 0.090 (0.066-0.113) | 0.062 (0.039-0.086) | 0.030 (0.006-0.053) | 0.036 (0.012-0.059) | 0.006 (0.003-0.008) | 16.67% | 0.5 |
| Age-OGTT0-  Hyperbilirubinemia | 0.090 (0.066-0.113) | 0.045 (0.021-0.069) | 0.033 (0.009-0.056) | 0.037 (0.013-0.060) | 0.006 (0.004-0.009) | 16.22% | 0.5 |
| Age-OGTT1-HDP | 0.136 (0.113-0.159) | 0.079 (0.056-0.103) | 0.072 (0.049-0.096) | 0.083 (0.059-0.107) | 0.011 (0.007-0.015) | 13.25% | 0.5 |
| Age-OGTT1-Preterm | 0.136 (0.113-0.159) | 0.043 (0.019-0.067) | 0.030 (0.006-0.054) | 0.036 (0.012-0.059) | 0.006 (0.003-0.009) | 16.67% | 0.5 |
| Age-OGTT1-  Hyperbilirubinemia | 0.136 (0.113-0.159) | 0.026 (0.002-0.049) | 0.033 (0.009-0.057) | 0.037 (0.013-0.060) | 0.003 (0.001-0.007) | 8.11% | 0.5 |
| Age-OGTT2-HDP | 0.197 (0.174-0.220) | 0.045 (0.021-0.069) | 0.074 (0.050-0.098) | 0.083 (0.059-0.106) | 0.009 (0.004-0.014) | 10.84% | 0.5 |
| Age-OGTT2-Preterm | 0.197 (0.175-0.220) | 0.048 (0.024-0.072) | 0.026 (0.002-0.050) | 0.035 (0.012-0.058) | 0.010 (0.005-0.015) | 28.57% | 0.5 |
